# Anti-Inflammatory, Antinociceptive, and LC-MS Metabolic Profile from *Pseudotrimezia juncifolia* (Klatt) Lovo & A. Gil

**DOI:** 10.3390/ph17081101

**Published:** 2024-08-22

**Authors:** Alan Silva Minho, Pamela Gomes de Almeida, Natália Naomi Kato, Ana Laura Macedo Brand, Roberto Fontes Vieira, Rafael Garrett, Norberto Peporine Lopes, Claudia Moraes Rezende, Patricia Dias Fernandes

**Affiliations:** 1 Laboratório de Farmacologia da Dor e da Inflamação, Instituto de Ciências Biomédicas, Universidade Federal do Rio de Janeiro, Rio de Janeiro 21941-902, Brazil; a_minho@me.com (A.S.M.); almeida_gp@outlook.com (P.G.d.A.); 2 NPPNS—Núcleo de Pesquisas em Produtos Naturais e Sintéticos, Faculdade de Ciências Farmacêuticas de Ribeirão Preto, Departamento de Ciências Biomoleculares, Universidade de São Paulo, Ribeirão Preto 14040-903, Brazil; nataliakato@usp.br (N.N.K.); npelopes@fcfrp.usp.br (N.P.L.); 3 Laboratório de Análise de Aromas, Instituto de Química, Universidade Federal do Rio de Janeiro, Rio de Janeiro 21941-901, Brazil; alaurambrand@gmail.com (A.L.M.B.); crezende@iq.ufrj.br (C.M.R.); 4 Embrapa Recursos Genéticos e Biotecnologia, Parque Estação Biológica, PqEB, Brasília 70770-901, Brazil; roberto.vieira@embrapa.br; 5 Laboratório de Metabolômica, Laboratório de Apoio ao Desenvolvimento Tecnológico (LADETEC), Instituto de Química, Universidade Federal do Rio de Janeiro, Rio de Janeiro 21941-901, Brazil; rafael_garrett@iq.ufrj.br

**Keywords:** *Pseudotrimezia juncifolia*, inflammation, pain, antinociception, dereplication, LC-MS, phenolics

## Abstract

*Pseudotrimezia juncifolia* (Klatt) Lovo & A. Gil (Iridaceae) is a popularly known species with primarily ornamental economic interest. It has traditional uses as purgative, in conditions related to the menstrual cycle, for blood purification, as wound healing, and as anti-inflammatory. The anti-inflammatory and antinociceptive activities of the decoction from its aerial stems, corms, and stamens are described here with dereplication studies on LC-MS/MS supported by the GNPS platform, where phenolic compounds were annotated and correlated with its biological activity. The decoction was evaluated in chemical (formalin and capsaicin) and thermal (hot plate) induced nociception or carrageenan-induced inflammation in mice. Decoction (at 10, 30, or 100 mg/kg doses) significantly reduced formalin- or capsaicin-induced nociception. All doses also demonstrated an antinociceptive effect in the hot plate model increasing the time the animal spent in responding to thermal signal. Naloxone partially reversed the antinociceptive effect. An anti-inflammatory effect was observed since a reduction in cell migration, protein extravasation interleukin-1, and tumor necrosis factor production induced by carrageenan in the subcutaneous air pouch was quantified. Metabolomic analyses showed a predominance of phenolic substances, mainly flavonoids and chlorogenic acids. The literature showed that these two groups have significant anti-inflammatory and analgesic activity, and chemical data corroborate the pharmacological results observed.

## 1. Introduction

Medicinal plants have been used by humanity for thousands of years. The secondary metabolites that make up these plants have contributed to the development of therapeutic medicines currently used in modern medicine, but the potential of higher plants as sources of new medicines remains a field to be widely explored [1].

Medicinal plants have, in their complex composition, several secondary metabolites with pharmaceutical uses, with around 40% of the medicines in use being obtained or inspired by these constituents. In several developing countries, herbal medicines are part of primary health care [2].

Among the most active metabolites are phenolic compounds, nitrogenous compounds from different skeletons, and terpenoids, due to their potential pharmacological properties. Inflammation, in turn, is the human body’s defense response to stimuli caused by allergens and/or injuries due to bacteria, viruses, fungi, physical agents, and immunological defects. On the other hand, uncontrolled inflammatory response causes problems such as allergies, cardiovascular dysfunctions, cancer, autoimmune diseases, and various metabolic syndromes, with a high negative impact, both economic and social. For this, extracts from medicinal plants have been reported, through several studies, as anti-inflammatory medicines around the world. With equal impact is the need for medicinal plants as analgesics, an ancient practice of humanity [3].

*Pseudotrimezia juncifolia* (Klatt) Lovo & A. Gil is an herbaceous plant from the Iridaceae family that occurs Brazilian *Cerrado*, described for its traditional medicine uses by different authors in local groups. The Scottish surgeon botanist George Gardner (1812–1849), one of the many naturalists who traveled throughout Brazil, revealed *P. juncifolia* (syn. *Trimezia juncifolia*) among the more than 6,000 species collected by him and described its fibrous root infusion as a powerful purgative, also active in the inflammation of brain membranes [4]. In 2012, Godinho studied *P. juncifolia* in terms of its external morphology aiming at a description of pharmacopeial microscopy due to its use, by traditional families from the city of Botucatu, São Paulo state, for blood purification, as wound healing and anti-inflammatory [5]. Yazbek et al. (2016), in a study on plants used during women’s health conditions among Brazilian cultures and based on the collection at the Ethnobotanical and Ethnopharmacological Center of the Federal University of São Paulo (1965 to 2012), described the popular use of *P. juncifolia* to restore menstrual flux to normal levels [6]. Despite its indications for popular medicine uses, the species remains unknown regarding the composition of its secondary metabolites. There are only a few studies related to the morphological and physicochemical characteristics of starches from *P. juncifolia* [7].

An alternative that has proven to be relevant for characterizing active extracts and avoiding re-isolation is the application of metabolomic techniques through hyphenated mass spectrometry and high-performance liquid chromatography [8]. The initial handy analysis was carried out by details characterization of the gas phase chemistry with a specific natural product [9], moving to the comparison of a small collection of spectra for the homolog series of a specific natural product moiety [10]. Nowadays, several computational tools can be applied, allowing the annotation of a large number of molecules from all classes of natural products [11].

In this study, the antinociceptive and anti-inflammatory activities of the decoction from the aerial stems, corms, and stamens of *P. juncifolia* were investigated, besides the dereplication studies carried out on LC-MS/MS supported by the GNPS platform [12], leading to the annotation of several phenolic compounds, of which some were correlated with biological activities.

## 2. Results

### 2.1. Nociception and Anti-Inflammatory Studies in P. juncifolia

#### 2.1.1. Decoction of *P. juncifolia* Does Not Present Toxic Effects

To investigate a possible toxic effect from decoction of *P. juncifolia*, analyses of possible alterations in mice behavior as well as in hematological parameters were performed. Animals received oral administration of *P. juncifolia* (at 100 mg/kg dose) and after 24 h were euthanized. No alteration occurred in blood parameters (i.e., platelet, leukocyte number, hematocrit, hemoglobin levels) or behavior.

#### 2.1.2. *P. juncifolia* Reduces Chemical-Induced Nociception

Intraplantar injection of formalin (2.5%) resulted in a licking response of paws in both phases of the model, 1st phase (0–5 min post-formalin injection) and 2nd phase (15–30 min post-formalin injection). Mice pretreated with vehicle resulted in 67.2 ± 12.8 s and 398.1 ± 81.5 s for each phase, respectively. Pretreatment of mice with morphine results in a 58% and 41.6% reduction in the response, respectively. Acetylsalicylic acid caused reductions of 30 and 46.4%, respectively.

**Figure 1:**
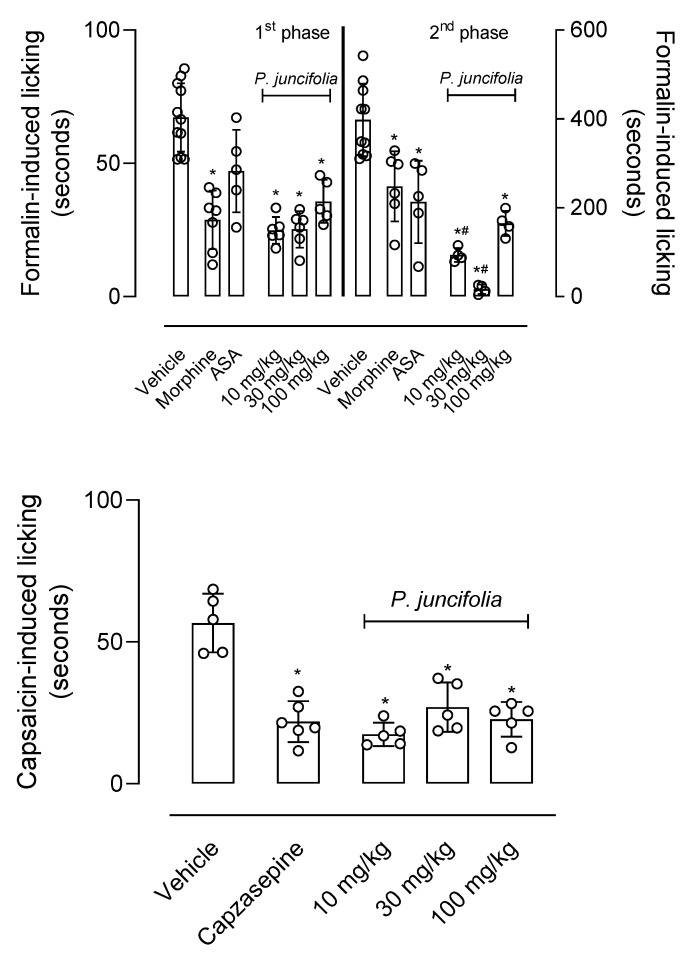
Decoction of *P. juncifolia* reduced nociception induced by formalin and capsaicin. Mice were pretreated with *P. juncifolia* (10, 30, or 100 mg/kg, oral), morphine (5 mg/kg, intraperitoneal), acetylsalicylic acid (200 mg/kg, oral), capsazepine (5 µg/kg, intraplantar) or vehicle 1 h after intraplantar injection of formalin (2.5%, 20 µL) or capsaicin (2 µg, 20 µL). The time the animal spent licking the injected paw was timed. Results are presented as media ± standard deviation (*n* = 6). Statistical analyses were performed with one ANOVA with Tukey post-test. * *p* < 0.05 when compared with vehicle-treated group and ^#^ *p* < 0.05 when compared with morphine- or ASA-treated group.

Oral administration of crescent (10–100 mg/kg) doses of *P. juncifolia* resulted in significant inhibition of the first phase in 30%, 62.5%, and 47%, respectively. It is interesting to note that all three doses of *P. juncifolia* significantly reduced the second phase (78.1%, 96.9% and 82.8%). Results were significant even when compared with morphine- or ASA-treated groups (Figure 1).

Figure 1 also shows the effects of *P. juncifolia* in the capsaicin-induced licking response. The positive control group was composed of animals pretreated with the capsaicin antagonist, capsazepine. This antagonist inhibited 61.3% of the licking effect caused by capsaicin. Any of the three doses of the decoction showed the ability to significantly reduce the algesic response to capsaicin. The observed inhibition was always greater than 55% at a dose of 30 mg/kg. It also draws attention to the fact that the dose of 10 mg/kg reduced nociception by almost 70%. It is interesting to note that all three doses showed an antinociceptive effect similar to that observed with capsazepine.

#### 2.1.3. *P. juncifolia* Reduces Thermal Nociception and Its Possible Mechanism of Action

**Figure 2:**
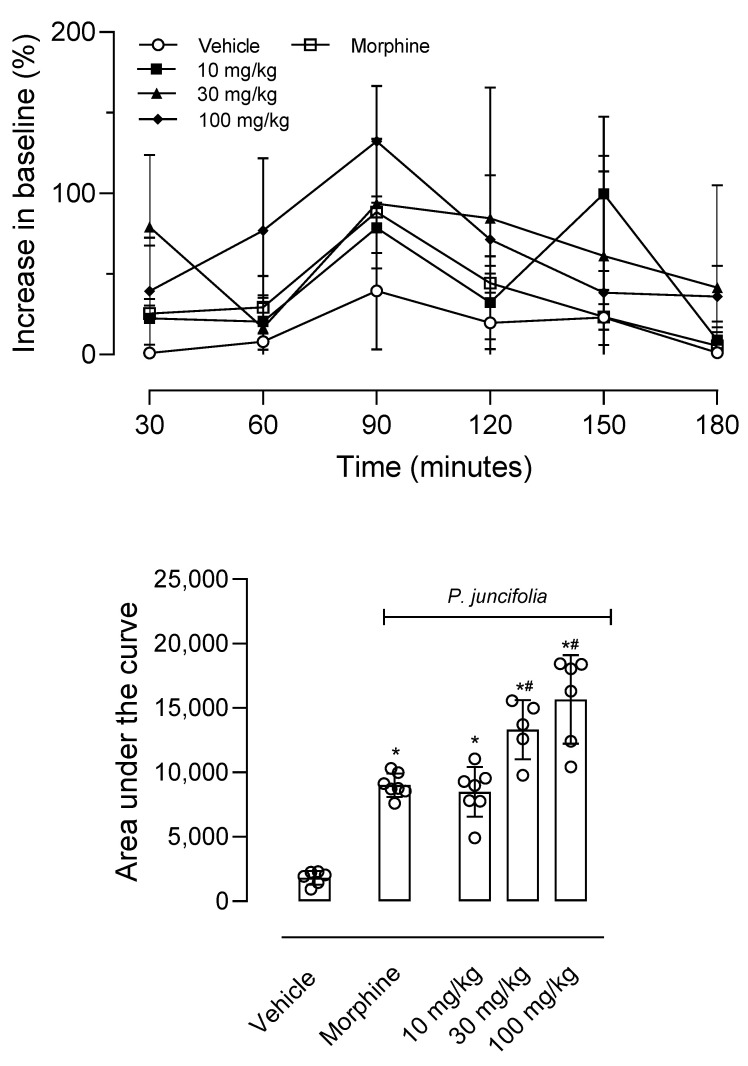
Decoction of *P. juncifolia* induces a central antinociceptive effect. Mice were pretreated with *P. juncifolia* (10, 30, or 100 mg/kg, oral), morphine (5 mg/kg, intraperitoneal), or vehicle and, every 30 min post-treatment, the response to the thermal injury was evaluated in the hot plate (55 ± 1 °C). Line graphs represent the time of response to the hot plate (shown as a percentage increase concerning the baseline) evaluated between 30 and 180 min after oral administration. The area under the curve was calculated based on data obtained from line graphs. The results are presented as mean ± SD (*n* = 8 per group) and statistical analyses were calculated by two-way ANOVA followed by Tukey’s post hoc test. * *p* < 0.05 when compared with the vehicle-treated group and # *p* < 0.05 when compared with the morphine-treated group.

A single dose of *P. juncifolia* increased the time of response to the thermal stimulus. The dose of 30 mg/kg resulted in a maximal effect at 90 min post-oral administration (100% increase in baseline) and until 180 min a significant effect could be observed. When mice were treated with the higher dose (100 mg/kg), an 80% increase in baseline was observed 60 min after administration. The maximal effect was reached at 90 min with a 125% increase in the baseline. When the data obtained over time were converted into an area under the curve (AUC) graph it was observed that, just like animals pretreated with morphine, animals that received increasing doses of decoction also had an increase in the AUC graph. It is noteworthy that both doses of 30 and 100 mg/kg could develop an antinociceptive response higher than that of morphine (Figure 2).

**Figure 3:**
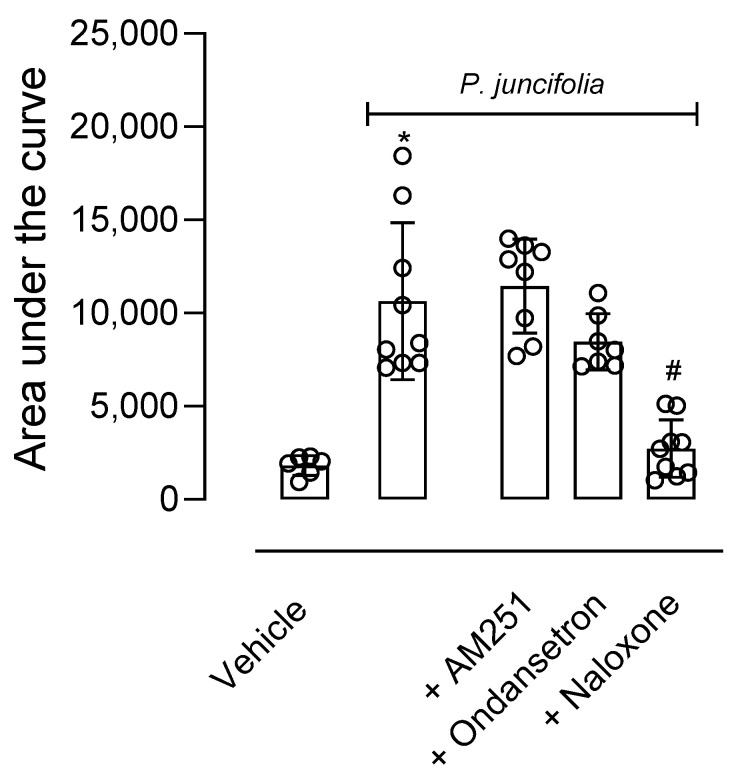
Effects of different antagonists on the antinociceptive activity of *P. juncifolia* evaluated in the hot plate model. Naloxone (1 mg/kg, i.p.), ondansetron (0.5 mg/kg, i.p.), or AM251 (1 mg/kg, i.p.) were administered 15 min before oral administration of *P. juncifolia* (100 mg/kg). Data are expressed as mean ± SD (*n* = 8). One-way ANOVA followed by Tukey’s post hoc test was used to calculate the statistical significance. * *p* < 0.05, when comparing *P. juncifolia*-treated mice to the vehicle-treated group, and # *p* < 0.05, when comparing antagonists pretreated mice with *P. juncifolia*-treated group.

As a significant antinociceptive effect was observed in the hot plate test, a possible mechanism of action of *P. juncifolia* decoction was investigated. In this regard, mice were pretreated with an opioid receptor antagonist (naloxone, 1 mg/kg, i.p.), a serotoninergic 5HT_3_ receptor antagonist (ondansetron, 0.5 mg/kg, i.p.), or a cannabinoid receptor antagonist (AM251, 1 mg/kg, i.p.). When injected alone, none of the antagonists developed an antinociceptive effect per se (F4,19 = 0.8138, *p* = 0.5320). As shown in Figure 3, the pretreatment of mice with naloxone 30 min before *P. juncifolia* (100 mg/kg) resulted in a complete blockage in the antinociceptive effect (*p* < 0.0001). When ondansetron or AM251 were administered, a reversion in the antinociceptive effect of *P. juncifolia* was not observed, even partially (Figure 3).

#### 2.1.4. *P. juncifolia* Presents an Anti-Inflammatory Effect

Then, it was decided to evaluate whether the plant’s decoction could also have an anti-inflammatory effect. In this sense, the animals were pretreated with increasing doses (10, 30, or 100 mg/kg) of *P. juncifolia* 1 h before the injection of carrageenan into the subcutaneous air pouch (SAP). After 24 h, the animals were euthanized, and the exudate was collected. The injection of carrageenan into the SAP leads to a 4.6-fold increase in the number of leukocytes that migrate to the cavity and the pretreatment of mice with dexamethasone reduced the cellular infiltration in 80%. Any of the doses (10, 30, or 100 mg/kg) of *P. juncifolia* significantly reduced cellular infiltrate by at least 70% (10 mg/kg reduced in 75.8%, 30 mg/kg reduced in 77.7% and 100 mg/kg reduced in 71.3%).

Another aspect of an inflammatory reaction is the increase in vascular permeability with consequent protein extravasation. Therefore, the quantification of the amount of protein extravasated into the exudate was performed. It can be observed that the carrageenan injection resulted in a 3.3-fold increase in the amount of protein accumulated in the exudate and the pretreatment with dexamethasone reduced this extravasation by 52.2%. The reduction observed after pretreatment with *P. juncifolia* resulted in a dose-dependent increase in inhibition (33.7%, 46.6%, and 53.1% to 10, 30 or 100 mg/kg, respectively).

**Figure 4:**
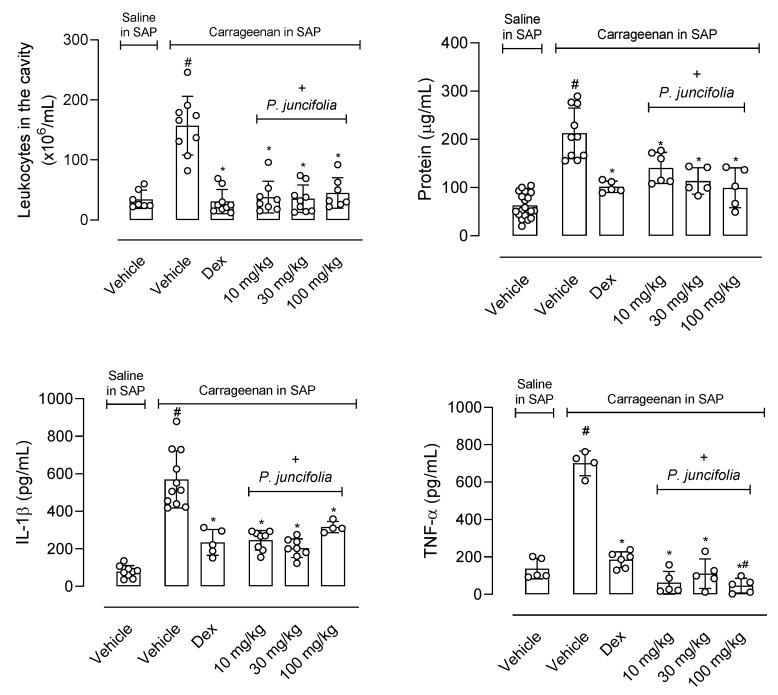
Effects of *P. juncifolia* extract in inflammation induced by carrageenan injection into the subcutaneous air pouch (SAP). Pretreatment of the animals with dexamethasone (1 mg/kg, i.p.) using *P. juncifolia* decoction (10, 30, or 100 mg/kg) or vehicle, 1 h before carrageenan injection into the SAP. The results are shown as the mean ± S.D. calculated with the Prism Software 8.02 (*n* = 6–8). One-way ANOVA was used followed by Tukey post hoc test to check the statistical significance. ^#^ indicates *p* < 0.05 when compared to the vehicle-treated group (animals treated with saline in the SAP) and * indicates *p* < 0.05 compared to the vehicle-treated group (animals that received carrageenan in the SAP).

We also decided to measure the amount of cytokine produced after carrageenan injection. The phlogistic agent induced a 7.2 and 5.1 increase in the production of IL-1β and TNF-α, respectively. The positive group, dexamethasone, reduced the amount of cytokine measured in the exudate by 58.9 and 73.6%, respectively. The pretreatment of all three doses of *P. juncifolia* resulted in a significant inhibition of both cytokines’ production. At least a 40% reduction was observed in cytokine production, and it is interesting to note that levels of TNF-α production were almost abolished by the highest dose (Figure 4).

It should be noted that, in all parameters evaluated, the inhibition caused by the plant decoction caused a similar or greater reduction than that observed when dexamethasone was used.

### 2.2. Metabolomic Analysis

**Figure 5:**
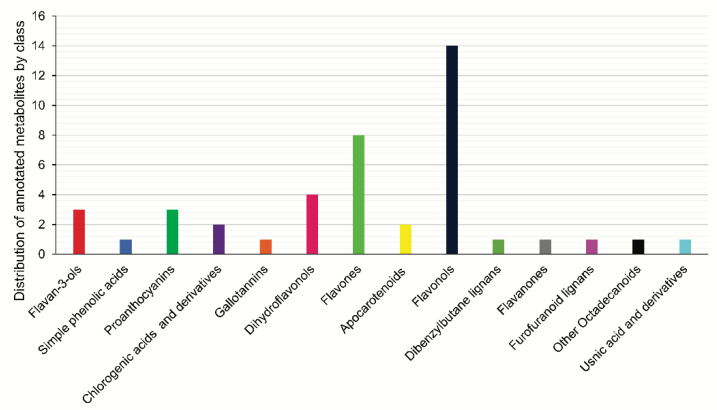
Analysis of the distribution of the annotated substances shows a clear predominance of flavonoids followed by proanthocyanins and chlorogenic acids and their derivatives.

**Figure 6:**
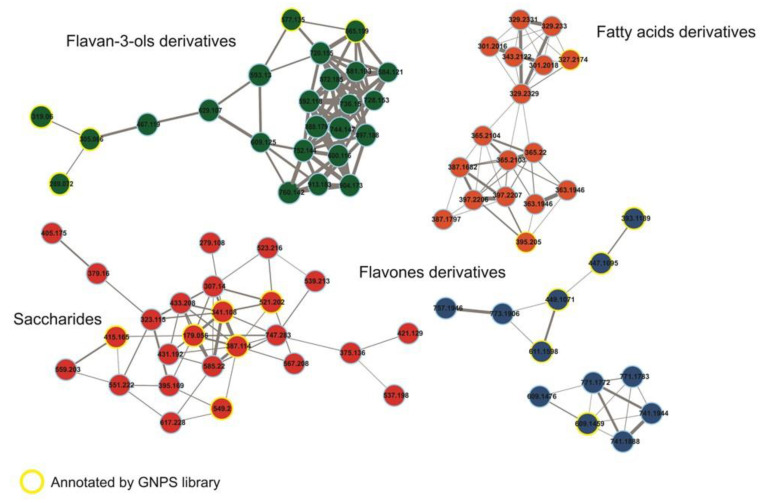
The main clusters of consensus MS/MS spectra representing the main secondary and primary metabolites of *P. juncifolia*. The molecular network was obtained using the Classic Molecular Network and the Feature-Based Molecular Network. The consensus MS/MS spectra are represented by the nodes. The weight of the edge, connecting the nodes, varies according to the cosine score.

**Figure 7:**
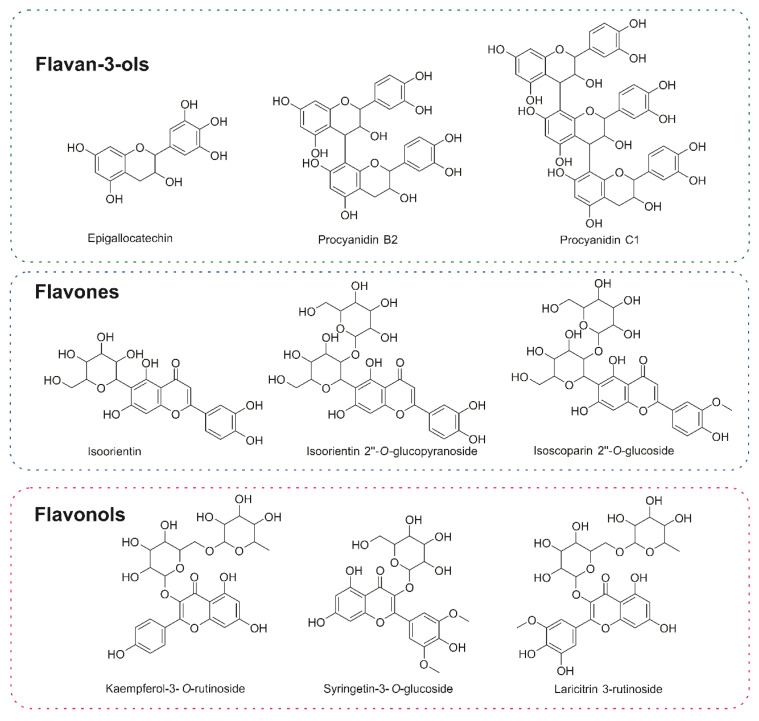
Chemical structures of flavonoid class annotated by GNPS library [12], which belong to the flavan-3-ol, flavones, and flavanol subclasses.

The forty-four compounds annotated by the GNPS library [12] and the analysis of the distribution of the secondary metabolites show a clear predominance of flavonoids followed by proanthocyanidins, chlorogenic acids, and their derivatives (Figure 5) (Table S1). The main secondary metabolites grouped in the molecular network are flavan-3-ols and flavones, in addition to the primary metabolites from the saccharides and fatty acids group (Figure 5). The classification of structures was according to the NP Classifier approach, associated with the GNPS. For the creation of the molecular network, the MS/MS data of each precursor ion were considered for the obtention of consensus spectra based on the spectral similarities and forming the clusters. In the case of the flavanol subclass, these are not grouped in the molecular network but present annotation of the precursor ions. Finally, all compounds have their molecular formula confirmed by HRMS (error less than 10 ppm) and, for each class, we manually inspected the gas phase chemistry involved in the fragmentation reactions (Figure 5, Figure 6 and Figure 7).

Most of the annotated secondary metabolites were glycoside flavonoids (Figure 5 and Figure 6), mainly linked through *O*-glycosidic bonds, resulting in the neutral sugar losses of 162u, corresponding to the *O*-hexoside group. Another representative group of secondary metabolites was chlorogenic acid and its analogs. The flavonoid–saccharide linkages need low collision energy to bond cleavage, which is common among the annotated flavonoid subclasses [13]. At the flavanol subclasses, we observed the loss of the 308u referring to the *O*-rutinose group linked to the C-3, consisting of the rhamnose and glucose. Finally, we combined molecular network analysis with rules for fragment reactions and chemotaxonomy to enhance the annotation of similar flavonoid glycoconjugates, as previously proposed by Pilon and co-workers [14]. For the chlorogenic acid, the classical neutral eliminations are in agreement with the hierarchical Clifford fragmentation scheme [15], confirming the structure of key compounds for the biological activity observed in this study.

## 3. Discussion

The decoction of the aerial stems, corms, and stamens of *P. juncifolia* revealed significant anti-inflammatory and antinociceptive effects. It was observed that the opioidergic pathway participates in the antinociceptive effect. An anti-inflammatory effect was also observed with leukocyte migration, cytokine production, and protein extravasation.

Here, we attempted to further characterize some of the mechanisms through which *P. juncifolia* exerts its antinociceptive effect. Pain is an endogenous physiological system that protects the body from potential damage. This phenomenon involves a multitude of events and systems such as oxidonitrergic, GABAergic, glutamatergic, opioidergic, cholinergic, serotonergic, adrenergic, and others. All these systems may together or alone interfere in different steps of the pain turning it into a complex phenomenon in such a way that alterations in one system can interfere with others [16,17]. A substance, whether natural or synthetic, can then act in one of several components to produce analgesia. Our data suggest that neither serotoninergic nor cholinergic pathways appear to be involved in the antinociceptive effect of decoction from *P. juncifolia,* since none of the antagonists used reversed the effects. Only naloxone, an opioid antagonist reverted the antinociceptive effect, demonstrating that the opioidergic system seems to be involved.

It could be seen that doses as low as 10 mg/kg significantly reduced the licking response induced by formalin injection in mice paws. The model of nociception induced by formalin raises a biphasic pattern with an initial phase (5 to 15 min post-injection) followed by a second phase (15 to 30 min post-injection) [18]. The first phase is associated with a direct activation of nociceptors while the second phase is associated with the release of inflammatory mediators that act in nociceptors and their local receptors [19]. The inhibitory effect observed to decoction in the second phase of this model also suggests that an anti-inflammatory effect may occur. These results led us to evaluate the ability of *P. juncifolia* to reduce the inflammatory phenomenon in another model. Thus, the model of leukocyte migration induced by the injection of carrageenan into the subcutaneous air pocket (SAP) was used. It is known that inflammation induced by carrageenan involves, in a complex way, the synthesis and/or release of several mediators and that, 24 h after carrageenan injection, there is a strong migration of leukocytes and an increase in countless inflammatory mediators [20].

The results verified through the SAP model complemented that obtained in the second phase of formalin-induced licking, as these models present an inflammatory profile that involves several inflammatory mediators. One hypothesis that can be raised is based on the reduction in the production and/or release of inflammatory substances involved in leukocyte chemotaxis. Therefore, the reduction in cell migration to the SAP may be due to the reduction in cytokine levels.

Some of the annotated compounds can be directly related to the observed biological activity report in the present paper. The first is the occurrence of chlorogenic acid and its analogs, which can be observed in different plants from *Cerrado* (Brazilian Savanna) with anti-inflammatory activity such as *Lychnophora* sp., popularly known as *Arnicas* [21], and *Annona nutans*, known as *Aracatia* [22], among other medicinal plants [23,24] and plants present in human diet [25]. In vivo studies showed significant antinociceptive activity in the acetic acid-induced mouse writhing test [26], inhibited carrageenan-induced paw edema and the number of flinches in the late phase of formalin-induced pain test, but chlorogenic acid did not inhibit the febrile response induced by lipopolysaccharide (LPS) in rats [27]. The anti-inflammatory mechanism of action was correlated with increasing prostaglandin E2 (PGE_2_) and alpha tumor necrosis factor (TNF-alpha) production by the cells and inhibiting monocyte chemoattractant protein-3 synthesis/release [28]. In this paper, the authors also observed the effects of *C*-beta-glucosyl apigenin on the production of PGE_2_ without altering the expression of cyclooxygenase 2 (COX-2) protein. Both results reinforce our present observations.

Finally, the major compounds reported in this article are the flavan-3-ol, flavones, and flavanol subclasses. These molecules can activate antioxidant pathways, leading to anti-inflammatory effects. Recently, Al-Khayri and co-workers [29] reported that flavonoids hinder the secretion of enzymes, like lysozymes and β-glucuronidase, as well as the release of arachidonic acid, thereby reducing inflammatory responses. Furthermore, they can modulate the expression and activation of cytokines, such as interleukin-1beta (IL-1β), interleukin-6 (IL-6), interleukin-8 (IL-8) and tumor necrosis factor-alpha (TNF-α). Additionally, gene expression of several pro-inflammatory modulators, such as nuclear factor kappa-light chain enhancer of activated B cells (NF-κB), intercellular adhesion molecule-1 (ICAM), vascular cell adhesion molecule-1 (VCAM), activator protein-1 (AP-1) and E-selectins, can be regulated. Flavonoids are also capable of inhibiting pro-inflammatory enzymes, like inducible nitric oxide (NO) synthase, lipoxygenase, and COX-2. Taken together, all these data supported by the literature [29,30] allowed us to correlate the chemical composition with the biological effects observed.

## 4. Materials and Methods

### 4.1. Plant Material and Lyophilized Preparation

*Pseudotrimezia juncifolia* (Klatt) Lovo & A. Gil. Det: B. M. T. was collected by Ismael da Silva Gomes on May 15 2018, at Brasília Botanical Garden—Caesb Trail, Brasília, DF, Brazil (15.880833 and −47.856944 WGS84) at an altitude of 1113 m, with a voucher deposited at the same botanical garden under the number CEN 120227.

Aerial stems, corms, and stamens of *P. juncifolia* were dried together under airflow at 37 °C for 48 h and all together reduced to a fine powder using a mill of knives. Dried material containing all parts (20 g) was mixed with 300 mL of boiling water. After overnight contact, the material was filtered and to the remaining powder 350 mL of aerial stems, corms, and stamens boiling water was added and the mixture stayed overnight. The whole procedure was repeated three times, and all filtered solutions were combined in a single preparation and submitted to lyophilization. Lyophilized material was stored at −20 °C. A stock solution was prepared at a concentration of 100 mg/mL. On the day of each assay, dilutions were prepared in distilled water and administered to animals at doses varying between 10 and 100 mg/kg, at a final volume of 0.1 mL.

### 4.2. MS Study

Liquid chromatography was carried out in a Dionex UltiMate 3000 UHPLC system (Thermo Scientific, Waltham, MA, USA) with a quaternary solvent delivery pump and a column oven compartment. Samples (4 μL) were injected using a TriPlus RSH Autosampler (Thermo Scientific) and separated on a reversed-phase column (Syncronis C_18_ 50 mm × 2.1 mm × 1.7 µm). The mobile phase consisted of ultrapure water (Milli-Q, Merck, Rahway, NJ, USA) (A) and acetonitrile (B), both containing 0.1% of formic acid. The separation was performed at an elution gradient of 5% B (0 min), 5–60% B (1–9 min), 60–98% B (9–13 min), 98% B (13–16 min), and 55% B (16.1–20 min) with a flow rate of 0.35 mL/min in an oven temperature at 40 °C. The Dionex UltiMate 3000 UHPLC system (Thermo Scientific) system was coupled to a QExactive Plus high-resolution mass spectrometer (Thermo Scientific) equipped with an electrospray ion source (ESI) that operated in both positive and negative ionization modes. The ionization parameters were spray voltage at 3.9 kV (+) and 3.6 (−); capillary temperature at 320 °C (+ and −); S-Lens level at 60 (+ and −); sheath gas 45 (+ and −) and auxiliary gas 15 (+ and −) (arbitrary units). The Full Scan analysis spanned over the *m/z* range of 100–1000 at a resolution of 35,000 (for both positive and negative modes), injection time of 180 ms and AGC 1 × 10^6^ for positive ion mode and injection time of 100 ms and AGC 3 × 10^6^ for negative ion mode. MS/MS data were acquired in both ionization modes using the ddMS2 experiment at a resolution of 17,500, injection time 50 ms, and AGC 1 × 10^5^ for positive and negative ion modes.

The LC-MS/MS data from the parts of *P. juncifolia* decoction were converted from Thermo .raw to .mzXML format using Proteowizard MSConvert before submission to the Global Natural Products Molecular Network (GNPS) for the annotation of compounds through library search based on the GNPS database [12]. The consensus MS/MS spectra were created by Classic Molecular Networking (MN) and Feature-Based Molecular Networking (FBMN). In the FBMN mode, we preprocessed the spectral data in the MZmine 2.53 to improve the feature detection and resolution of isomers. In both analyses, the parameters set in the GNPS platform were Precursor ion 0.02 Da, Fragment ion 0.02 Da to the annotation of precursor ion and, for the connection of consensus MS/MS spectra, cosine score above 0.60 and more than 4 matched fragments peaks were used. Hand inspections were performed based on the gas phase chemical rules [31].

### 4.3. Animals

Swiss Webster mice (25–30 g) were donated by Instituto Vital Brazil (Niterói, Rio de Janeiro, Brazil) and maintained in a room where a 12-h light–dark cycle was established, at a temperature of 22 ± 2 °C and humidity from 60% to 80%, with food and water provided ad libitum. One hour before the experiments, the animals were acclimatized to laboratory conditions and were treated only once throughout the experiments. The protocols for all experiments were in accordance with the Guidelines on Ethical Standards for Investigating Experimental Pain in Animals [32]. Likewise, the principles and guidelines adopted by the National Council for the Control of Animal Experimentation (CONCEA), approved by the Ethics Committee for Animal Research Committee (#31/19, 34/19, 28/20) were followed. The experimental protocols were all performed during the light phase. The number of animals used was kept to a minimum and the number of animals in each experiment is cited in the legend of the Figure that corresponds to the experiment performed. At the end of each experiment, the animals were killed by an overdose of ketamine/xylazine.

### 4.4. Reagents and Laboratories Supplies

Ammonium formate, acetylsalicylic acid (ASA), capsaicin, acetonitrile, capsazepine, dexamethasone, and acetylsalicylic acid were purchased from Sigma-Aldrich (St. Louis, MO, USA). Ethanol and formalin were purchased from Merck Inc. (Rio de Janeiro City, Rio de Janeiro, Brazil). Morphine sulfate was kindly provided by Cristália (São Paulo, Brazil). Cytokine kits were obtained from B&D Drugs and dissolved in phosphate buffer saline (PBS) before use. Capsaicin was dissolved in 80% (*v*/*v*) ethanol plus 20% PBS. All drugs were diluted just before their use.

### 4.5. Administration of Pseudotrimezia juncifolia and Drugs

The decoction of the aerial stems, corms, and stamens of *P. juncifolia* was dissolved in distilled water for the preparation of 100 mg/mL stock solutions. The solutions for pharmacological use were freshly prepared. Doses of 10–100 mg/kg (final volume of 0.1 mL per animal) were administered by oral gavage. The reference drugs were acetylsalicylic acid (ASA, 200 mg/kg) and morphine (5 mg/kg). The dose of AAS and morphine used here was based on previous studies of the group, where its ED50 was calculated, i.e., the dose that caused a 50% reduction in the nociceptive effect. The control group was given a vehicle only.

### 4.6. In Vivo Toxicity Test

For different groups of animals, 100 mg/kg of *P. juncifolia* decoction was administered orally. After 24 h of administration, mice were sacrificed with ketamine (50 mg/kg of xylazine (20 mg/kg). Then, a blood sample was collected in a heparinized tube. The femur was removed and the bone marrow from each femur was washed with 1 mL of saline solution (0.9% NaCl) with heparin. Both bone marrow and blood samples were subjected to complete blood count and cell count using an automatic cell counter (PocH-100iV Diff, Sysmex, Kobe, Japan).

### 4.7. Formalin-Induced Nociception

This assay was performed as described by Sakurada et al. (1998) and adapted by Matheus et al. [33,34]. This model is characterized by a response that occurred in two phases. The first phase (acute neurogenic pain) occurred during the first 5 min after the intraplantar injection of formalin and the second phase (inflammatory pain) occurred during the 15 to 30 min post-injection. Twenty μL of formalin (2.5% *v*/*v*) was introduced onto the dorsal surface of the animals’ left hind paw and the time the animals spent licking the injected paw was immediately recorded. Oral doses of *P. juncifolia* decoction, morphine, ASA, or vehicle were administered to the animals 60 min before formalin administration.

### 4.8. Capsaicin-Induced Nociception

These experimental protocols were performed as described by Sakurada et al. (1998) [33]. Mice received an intraplantar injection of capsaicin (20 μL, 2 μg/paw). Immediately after the injection, the animals were placed individually in a transparent box and the time that the animal kept licking or biting the capsaicin-injected paw was recorded for 5 min and considered to be the nociceptive reaction. The animals were pretreated 60 min before the intraplantar injection of capsaicin with *P. juncifolia* decoction (10–100 mg/kg, p.o.), capsazepine (capsaicin receptor antagonist, 10 μg/paw) or vehicle (p.o.).

### 4.9. Thermal-Induced Nociception (Hot Plate Test) and Mechanism of Action

Mice were tested according to the method described by Sahley and Berntson (1979) and adapted by Matheus et al. [34,35]. Mice were placed on a hot plate (Insight Equipment, São Paulo, Brazil) set at 55 ± 1 °C. The reaction time (licking of paws or jumping) was recorded every 30 min post oral administration of *P. juncifolia* decoction, vehicle, or morphine until 180 min. The animals’ average reaction time (in seconds) obtained at 30 and 60 min before oral administration was basal (normal reaction to temperature). The area under the curve (AUC) plots was calculated from time course plots. The formula employed was based on the trapezoid rule and used to calculate the AUC, where AUC = 30 × IB ((min 30) + (min 60) + (min 180)/2), with IB being the increase from baseline (in%). Treatments were administered (one each) i.p. 30 min before administration of *P. juncifolia* (100 mg/kg, p.o.), AM251 (cannabinoid CB1 receptor antagonist, 1 mg/kg), naloxone (opioid receptor antagonist, 1 mg/kg) or ondansetron (serotonergic 5-HT3 receptor antagonist, 0.5 mg/kg). The antinociceptive effect was evaluated in the hot plate test.

### 4.10. Carrageenan-Induced Leukocyte Migration into the Subcutaneous Air Pouch (SAP)

In accordance with Raymundo et al. (2011) [20], the animals were given a subcutaneous injection of 10 mL of sterile air in the dorsal region on three alternate days, as well as an injection of sterile carrageenan suspension (1%; 1 mL) in the SAP. The mice were divided into a group treated with vehicle, *P. juncifolia* decoction, or dexamethasone 1 h before carrageenan injection, and a group treated with vehicle and receiving PBS (phosphate buffered saline, 1 mL) in SAP. After 24 h, all mice were sacrificed. The SAP was further washed with 1 mL of sterile PBS and the collected exudates were centrifuged (5000× *g*, 10 min, 4 °C) and the supernatant aliquots were stored at −20 °C until the start of the assays.

### 4.11. Quantification of TNF-α, IL-1β, IFN-γ and Protein

The supernatants that were obtained from the exudates collected from the SAP were used to measure tumor necrosis factor-α (TNF-α) and interleukin 1β (IL-1β). Enzyme-linked immunosorbent assay (ELISA), according to the manufacturer’s instructions (B&D, Macon, GA, USA), was used. The BCA method (BCA™ Protein Assay Kit, Pierce, Thermo Scientific) was used to measure extravasated protein.

### 4.12. Statistical Analysis

For each experiment, the number of animals used was indicated in the legend of each Figure. Results are reported as mean ± SD, calculated using Prism Software 8.02 (GraphPad Software, La Jolla, CA, USA). One-way or two-way analysis of variance (ANOVA) was followed by Tukey’s post hoc test and employed for unpaired data in cases where more than two groups were compared to the equally employed control. In cases where F reached the necessary level of statistical significance, post hoc tests were performed. When *p* was less than 0.05, differences between groups were considered significant.

## 5. Conclusions

*P. juncifolia* decoction, at doses of 10, 30, or 100 mg/kg, significantly reduced nociception induced by formalin or capsaicin and in the hot plate model. Naloxone partially reversed the antinociceptive effect. The anti-inflammatory effect was observed with the reduction in cell migration, protein extravasation, and the production of interleukin-1 and tumor necrosis factor, induced by carrageenan in the subcutaneous air pouch, concluding that *P. juncifolia* exhibits significant anti-inflammatory and antinociceptive activity, reinforcing its popular use. Chemical studies reveal the predominance of phenolic compounds (especially flavonoids and chlorogenic acid derivatives) and, by correlating the annotated structures with the literature review, it was possible to suggest and discuss the potential mechanisms of action involved in the conducted assays.

It is important to observe that interactions between phytochemicals can be additive, synergistic, or antagonistic. As demonstrated by Wang et al. [36], combinations of flavonoids and non-flavonoids can be statistically synergistic and antagonistic, in this study regarding their antioxidant activities using the DPPH· assay. In addition to the antioxidant capacity, important roles in the inflammation process, the increase in bioavailability, the interaction with the intestinal microbiome, and the action on different signaling pathways, are important aspects for understanding the synergistic anti-inflammatory effects in cells, animals, and humans.

When comparing the effects of *P. juncifolia* decoction with non-steroidal (NSAIDs) or steroidal anti-inflammatory drugs (SAIDs) on the market, we can infer that *P. juncifolia* decoction did not show adverse effects in the models evaluated. NSAIDs have several adverse effects that limit their use, such as reduced gastric protection, reduced renal blood flow (which may lead to kidney injury and nephrotoxicity), and reduced platelet aggregation, among others. SAIDs produce several systemic and metabolic changes, such as immunosuppression, fluid retention, and Cushing’s syndrome, among others. All side effects can be serious and limit or prohibit the use of these medicines. Therefore, the use of *P. juncifolia* decoction may become a promising alternative for patients who cannot use traditional medicines.

## Data Availability

The original contributions presented in the study are included in the article/Supplementary Material, further inquiries can be directed to the corresponding author.

## Supplementary Materials

The following supporting information can be downloaded at: https://www.mdpi.com/article/10.3390/ph17081101/s1, Table S1. Chemical composition. retention times and *m*/*z* data by UHPLC–MS in a reversed-phase column (Syncronis C_18_) analysis for *P. juncifolia* aerial stems, corms and stamens. based on GNPS library [12].
