# Supplementary material for: Anti-Inflammatory, Antinociceptive, and LC-MS Metabolic Profile from Pseudotrimezia juncifolia (Klatt) Lovo & A. Gil
Source: Pharmaceuticals (Basel). 2024 Aug 22;17(8):1101. doi: 10.3390/ph17081101 (PMC11359133; doi:10.3390/ph17081101)
Supplement: Supplementary file 1 [file pharmaceuticals-17-01101-s001.zip › pharmaceuticals-3138680-supplementary.pdf]

Table S1 – Chemical composition. retention times and *m/z* data by UHPLC–MS in a reversed-phase column (Syncronis C_{18}) analysis for *P. juncifolia* aerial stems. corms and stamens. based on GNPS library [12]

No.	RT	Compound	Molecular Formula	Adduct	*m/z*	MS/MS	Error (ppm)
				[M-H]-	305.0664	261; 219; 179; 167; 137; 125	0.98
1	2.54	Epigallocatechin	$C_{15}H_{14}O_7$	[M+H]+	307.0789	289; 271; 247; 223; 205; 205; 195; 181; 169;163; 151; 139; 123	-0.98
2	4.86	Phaseoloidin	$C_{14}H_{18}O_9$	[M-H]-	329.0865	269; 209; 181; 167; 123; 113; 101; 89; 71; 59	-0.61
3	5.09	Procyanidin B1	$C_{30}H_{26}O_{12}$	[M-H]-	577.134	407; 289; 161; 125; 109	1.73
				[M-H]-	577.134	441; 407; 345; 289; 281; 255; 245; 203; 179; 161; 151; 125; 109	
4	5.49	Procyanidin B2	$C_{30}H_{26}O_{12}$	[M+H]+	579.1500	451; 427; 409; 287; 257; 247; 163; 139; 127; 107	-1.21
				[M-H]-	289.0706	271; 245; 227; 221; 203; 179; 151; 125; 109	2.42
5	5.7	Catechin	$C_{15}H_{14}O_6$	[M+H]+	291.0863	207; 189; 179; 165; 147; 139; 123	-0.69
6	5.9	Procyanidin C1	$C_{45}H_{38}O_{18}$	[M-H]-	865.1974	407; 289; 161; 125	1.85
7	5.98	5-O-Caffeoylquinic acid	$C_{16}H_{18}O_9$	[M-H]-	353.088	191; 179; 135	5.61
8	6.34	1.6-Di-*O*-galloyl-β-D-glucose	$C_{20}H_{20}O_{14}$	[M-H]-	483.0769	331; 313; 271; 241; 211; 169; 125	-7.04
9	6.54	(+)-Dihydromyricetin	$C_{15}H_{12}O_8$	[M-H]-	319.0448	301; 257; 193; 175; 165; 125	3.76
10	6.65	*trans*-4-*O*-Caffeoylquinic acid	$C_{16}H_{18}O_9$	[M-H]-	353.0866	191; 179; 173; 135	-0.28
11	7.15	(+)-Catechin	$C_{15}H_{14}O_6$	[M+H]+	291.0862	207; 189; 179; 165; 147; 139; 123	-0.34
12	7.35	6.8-Di-*C*-β-glucosylluteolin	$C_{27}H_{30}O_{16}$	[M-H]-	609.1434	593; 503; 473; 383; 353;325(-28u); 297	-2.63

No.	RT	Compound	Molecular Formula	Adduct	*m/z*	MS/MS	Error (ppm)
13	7.74	6.8-Di-D-galactosylapigenin	$C_{27}H_{30}O_{15}$	[M-H]-	593.1502	503; 473; 383; 353; 325; 297	0.34
14	8.09	Luteolin	$C_{15}H_{10}O_{6}$	[M-H]-	285.0399	241; 217; 199; 175	2.10
				[M-H]-	303.0499	285; 259; 175; 125	4.29
15	8.1	Taxifolin	$C_{15}H_{12}O_{7}$	[M+H]+	305.0655	287; 259; 231; 195; 153; 149; 123	-2.62
16	8.25	Loliolide	$C_{11}H_{16}O_{3}$	[M+H]+	197.117	161; 151; 135; 107	-1.01
17	8.27	Isoorientin 2′′-*O*-glucoside	$C_{27}H_{30}O_{16}$	[M+H]+	611.1590	449; 383; 353; 329; 299	-2.62
				[M-H]-	447.0928	357; 327; 299; 285	1.57
18	8.42	Isoorientin	$C_{21}H_{20}O_{11}$	[M+H]+	449.1057	431; 413; 395; 353; 329; 299	-4.68
19	8.48	Myricetin 3-*O*-glucoside	$C_{21}H_{20}O_{13}$	[M-H]-	479.0813	317; 287; 271	-1.46
20	8.51	Myricetin-3-*O*-rutinoside	$C_{27}H_{30}O_{17}$	[M-H]-	625.1406	317; 287; 271; 179; 151	1.12
21	8.82	Flavosativaside	$C_{27}H_{30}O_{15}$	[M-H]-	593.1465	413; 323; 311; 293	-5.90
22	8.89	Clitorin	$C_{33}H_{40}O_{19}$	[M-H]-	739.2057	283; 255; 227; 179; 167	-3.11
23	8.94	Dihydrokaempferol	$C_{15}H_{12}O_{6}$	[M-H]-	287.056	259; 243; 177; 151; 125	3.48
24	9.00	Isoscoparin-2′′*O*-glucoside	$C_{28}H_{32}O_{16}$	[M-H]-	623.161	443; 323	0.64
25	9.17	Dihydrokaempferol	$C_{15}H_{12}O_{6}$	[M-H]-	287.0554	269; 259; 243; 201; 151; 125	1.39
26	9.19	Isorhamentin 3-*O*-β-D-rutinoside	$C_{28}H_{32}O_{16}$	[M-H]-	623.1611	315; 299; 271	0.80
27	9.23	Rutin	$C_{27}H_{30}O_{16}$	[M-H]-	609.147	427; 343; 299; 271; 255; 243; 179; 151	3.28
28	9.23	Quercetin 3-glucoside	$C_{21}H_{20}O_{12}$	[M-H]-	463.0867	315; 299; 271; 255; 243	-0.86
29	9.37	methoxy-myricetin-3-O-hexoside	$C_{22}H_{22}O_{13}$	[M-H]-	493.097	329; 315; 287; 271; 259	-1.22
30	9.46	Laricitrin 3-rutinoside	$C_{28}H_{32}O_{17}$	[M-H]-	639.1606	329; 315; 287; 271; 179;	7.98
31	9.60	Secoisolariciresinol	$C_{20}H_{26}O_{6}$	M-2H2O+H	327.1584	295; 163; 151; 137	-1.83
32	9.68	Kaempferol 3-*O*-rutinoside	$C_{27}H_{30}O_{15}$	[M-H]-	593.15	283; 255; 227; 151	0.00
33	9.78	Byzantionoside B	$C_{19}H_{32}O_{7}$	[M+H]+	373.222	211; 193; 175; 135; 109; 95	0.00

No.	RT	Compound	Molecular Formula	Adduct	*m/z*	MS/MS	Error (ppm)
34	9.85	Kaemferol 3-*O*-glucopyranoside	C21H20O11	[M-H]-	447.0921	283; 255; 227; 179	1.79
35	9.88	Kaempferol 3-*O*-rutinoside	C27H30O15	[M-H]-	593.15	285; 255; 227; 151	2.19
36	9.88	(*S*)-2.3-Dihydroluteolin	C15H12O6	[M-H]-	287.055	227; 151; 135; 125; 107	-3.83
37	9.91	5.7-dihydroxy-6-methoxy-2-[4-[(2*S*.3R.4S.5*S*.6*R*)-3.4.5-trihydroxy-6-(hydroxymethyl)oxan-2-yl]oxyphenyl]chromen-4-one	C22H22O11	[M-H]-	461.1078	297; 283; 255; 163	1.95
38	10.02	Kaempferol 3-*O*-α-L-arabinopyranoside	C20H18O10	[M-H]-	417.0816	283; 255; 227	0.96
39	10.08	Isorhamentin 3-*O*-β-D-rutinoside	C28H32O16	[M-H]-	623.1606	357; 315; 299; 271; 243	2.09
40	10.10	Syringetin-3-*O*-glucoside	C23H24O13	[M-H]-	507.1131	343; 329; 301; 287; 273; 169; 151; 125	1.38
41	10.15	Phenol. 3.3′-[(1*R*.3a*S*.4*R*.6a*S*)-tetrahydro-1*H*.3*H*-furo[3.4-*c*]furan-1.4-diyl]bis[6-methoxy-. *rel*-(9CI. ACI)	C20H22O6	M-H2O+H	359.1489	341; 323; 291; 269; 187; 137	-1.47
42	12.33	(10E.15E)-9.12.13-trihydroxyoctadeca-10.15-dienoic acid	C18H32O5	[M-H]-	327.2172	309; 291; 229; 211; 171	1.83
43	14.40	Usnic acid	C18H16O7	[M-H+CH3OH]	343.0815	299; 259; 255; 231; 83	0.87
